# Preparation of Two Types of Polymeric Micelles Based on Poly(β-L-Malic Acid) for Antitumor Drug Delivery

**DOI:** 10.1371/journal.pone.0162607

**Published:** 2016-09-20

**Authors:** Tiehong Yang, Wei Li, Xiao Duan, Lin Zhu, Li Fan, Youbei Qiao, Hong Wu

**Affiliations:** 1 Department of Pharmaceutical Analysis, School of Pharmacy, Fourth Military Medical University, Xi’an, China; 2 Department of Pharmaceutical Sciences, Irma Lerma Rangel College of Pharmacy, Texas A&M University Health Science Center, Kingsville, Texas, 78363, United States; Helsingin Yliopisto, FINLAND

## Abstract

Polymeric micelles represent an effective delivery system for poorly water-soluble anticancer drugs. In this work, two types of CPT-conjugated polymers were synthesized based on poly(β-L-malic acid) (PMLA) derivatives. Folic acid (FA) was introduced into the polymers as tumor targeting group. The micellization behaviors of these polymers and antitumor activity of different self-assembled micelles were investigated. Results indicate that poly(ethylene glycol)-poly(β-L-malic acid)-campotothecin-I (PEG-PMLA-CPT-I, P1) is a grafted copolymer, and could form star micelles in aqueous solution with a diameter of about 97 nm, also that PEG-PMLA-CPT-II (P2) is an amphiphilic block copolymer, and could form crew cut micelles with a diameter of about 76 nm. Both P1 and P2 micelles could improve the cellular uptake of CPT, especially the FA-modified micelles, while P2 micelles showed higher stability, higher drug loading efficiency, smaller size, and slower drug release rate than that of P1 micelles. These results suggested that the P2 (crew cut) micelles possess better stability than that of the P1 (star) micelles and might be a potential drug delivery system for cancer therapy.

## Introduction

As drug carriers, biomacromolecules have attracted significant interest in recent years due to their renewability, low toxicity, biocompatibility, and biodegradability[1–4]. Moreover, the abundant reactive functional groups of biomacromolecules, such as hydroxyl, amino and carboxyl, are readily available for linking various bioactive molecules (drugs, ligands, antibodies, etc.). Among various biomacromolecules, polysaccharides[3,4] (chitosan, sodium alginate, cyclodextrin, pectin, etc.) and proteins[2] (gelatin, albumin, fibroin, etc.) are currently the most commonly used in drug delivery.

Poly(β-L-malic acid) (PMLA) is a natural aliphatic polyester obtained from the microorganism Physarum polycephalum[5], it could degrade into malic acid firstly and then degrades into carbon dioxide and water by tricarboxylic acid cycle in vivo[6,7]. As a novel drug delivery carrier, PMLA is superior to polysaccharides and proteins due to its biodegradable, non-toxic and non-immunogenic properties[8,9], especially its numerous pendent carboxyl groups which allow chemotherapeutics, targeting ligands and other functional groups to decorate easily on the same polymer backbone[10]. “Polycefin”[11–14], a new prototype of a multifunctional nanocarrier based on PMLA, was synthesized for targeted delivery of antisense oligonucleotides/drugs and monoclonal antibodies to tumor cells. However, few applications were reported due to difficulties in preparation, and to some extent, the high water-solubility of PMLA. In our previous study[15], the synthesis process of PMLA was optimized and PMLA with definite molecular weight for the application of drug delivery carriers were obtained. In addition, it was reported that introducing hydrophobic groups via covalent bonds to PMLA scaffold could reduce its water-solubility[16]. Water-insoluble drugs could be used as the hydrophobic groups to reduce water-solubility of PMLA, and the solubility of the drugs itself could be tremendously improved at the same time.

Camptothecin (CPT), a DNA-toxin, inhibits topoisomerase I that is involved in DNA replication to induce cell apoptosis[17]. However, it hasn’t been widely used in clinic because of its poor aqueous solubility and low therapeutic index. Moreover, CPT exists in two forms at different pH conditions, the active closed lactone ring form and the inactive carboxylate form. At alkaline and physiological conditions, the lactone ring is unstable and rapidly converted to the inactive carboxylate form, resulting in inactivity[18]. Therefore, various polymeric carriers have been used to improve solubility, stability and reduce the renal clearance of CPT[19,20].

Polymeric micelles (PMs) formed by the self-assembly of amphiphilic copolymers are promising nanocarriers for drug delivery[21–23]. PMs have some advantages compared with polymer-drug conjugates. For example, hydrophobic drugs could be encapsulated in internal cavity of polymeric micelles to improve its solubility and stability. In addition, a single polymer-drug conjugate could combine only one ligand molecule, while a PM molecule could combine multiple ligands which effectively enhance targeting and endocytosis. In our previous research[15], CPT-PMLA conjugate was synthesized by conjugating CPT to PMLA scaffold via ester bond. The CPT-PMLA conjugate could form micelles when the graft ratio of CPT ranged from 5 wt% to 8 wt%. However, the formed micelles were unstable and the CPT was easily hydrolyzed.

**Fig 1:**
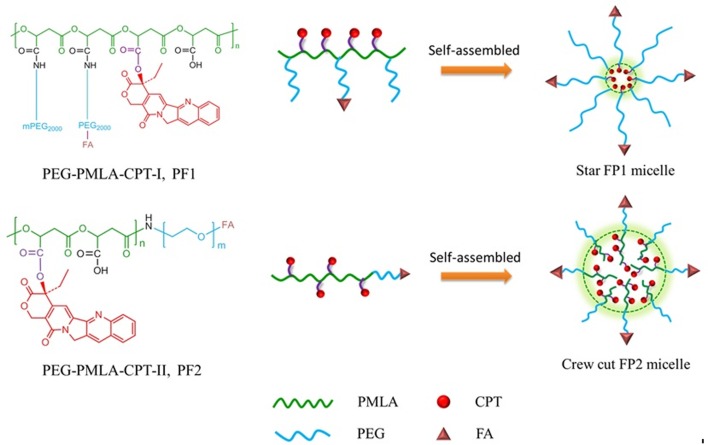
Two types of self-assembled PMs based on CPT-conjugated PMLA.

In this paper, to improve the micellization ability and stability of CPT-PMLA conjugate, PEG was introduced into the CPT-PMLA conjugate as a protective hydrophilic shell in two different connection ways. In one way, PEG was located inside the PMLA scaffold and formed grafted copolymers (P1). In another way, PEG was connected to the terminal of PMLA scaffold and formed amphiphilic block copolymer (P2). In the process of self-assembled micelles formation, P1 was expected to form star micelles in aqueous solution, for the hydrophilic block PEG (staying outside) longer than hydrophilic CPT (staying inside). Whereas P2 was expected to form crew cut micelles, for the polymer with the hydrophobic block (CPT-PLMA) longer than the hydrophilic one (PEG) [24,25] (Fig 1). The micellization behaviors of the polymers and antitumor activity of different self-assembled micelles were also compared to obtain a polymer micelle with high drug loading efficiency and good stability. Since PMLA was negatively charged and was unfavorable for cell endocytosis, P1 and P2 micelles were functionalized with folic acid (FA) moieties as a ligand to enhance cellular uptake and target selectivity of tumor cells.

## Materials and Methods

### Materials

mPEG_2000_-NH_2_, FA-PEG_2000_-NH_2_, FITC-PEG_2000_-NH_2_ were purchased from American Nanocs Inc. L-aspartic acid, N-hydroxysuccinimide (NHS), 1-ethyl-3-(3-dimethylaminopropyl) carbodiimide (EDC), 4-dimethylamiopryidine (DMAP), tetraethyl ammonium hydroxide, trifluoroacetic anhydride (TFAA), propidium iodide (PI), camptothecin (CPT), 3-(4,5-Dimethylthiazol-2-yl)- 2,5-diphenyltetrazolium bromide (MTT) and fluorescein isothiocyanate (FITC) were purchased from Sigma Aldrich. NaBr, NaNO_2_, ethyl acetate, ethyl ether, acetone, dimethylsulfoxide (DMSO), 1,4-dioxane, benzyl alcohol and benzoic acid were purchased from Tianjin Fuchen Inc. RPMI-1640 culture medium and fetal bovine serum (FBS) were obtained from GIBCO. Phosphate buffered saline (PBS) was purchased from KeHao BioEngineering CO. LTD and was adjusted to pH 5.0 with 0.1 mol/L HCl.

### Synthesis of poly (benzyl malate) (PMLABz)

PMLABz, with a molecular weight of 16 kDa, was synthesized by a ring-opening polymerization method according to a previously reported improved design[26]. Firstly, RS-4-benzyloxycarbonyl- 2-oxetanone (MLABz) was synthesized. In brief, L-aspartic acid was diazotized to obtain bromosuccinic acid. Next, bromosuccinic acid anhydride was produced by dehydration of bromosuccinic acid with trifluoroacetic anhydride (TFAA) as catalyst. Benzyl alcohol was then mixed with the acid anhydride to give a monoester mixture, transformed into MLABz in the weak alkali condition of pH 7.5. Secondly, PMLABz was synthesized. Briefly, 320 μL of an ethanol solution of tetraethylammonium benzoate (8×10^−3^ mol/L) was placed in the polymerization flask, the alcohol was removed under vacuum at room temperature (RT). 4 g of MLABz was transferred into the polymerization flask containing the initiator under nitrogen. The polymerization was conducted under vacuum at 40°C for 3 days (disappearance of the lactone peak at 1825 cm^-1^ from the IR spectroscopy). The polymer was dissolved in acetone, precipitated in ethanol and dried under vacuum for 24 h.

### Synthesis of PMLA

PMLABz (2 g) was dissolved in 20 mL of 1,4-dioxane with Pd/C catalyst (0.2 g) and hydrogenated at room temperatures (RT). After complete reaction, the catalyst was filtered and 1,4-dioxane was removed by rotary evaporation. The polymer was then dissolved in small amount of 1,4-dioxane, precipitated in abundance of diethyl ether and dried under vacuum for 24 h.

### Synthesis of PEG-PMLA-CPT-I (P1)

PMLA (116 mg, 1 mmol with regard to malyl units), CPT (0.2 mmol), EDC (0.3 mmol) and DMAP (0.02 mmol) were mixed in 15 mL of DMSO, stirring at RT for 12 h. The solution was dialyzed (MWCO: 3.5 kDa) against DMSO for 24 h to remove unreacted CPT and other small molecules, then dialyzed (MWCO: 3.5 kDa) against deionized water for 24 h at 4°C. Finally, the solution was lyophilized to give PMLA-CPT as khaki powder.

PMLA-CPT (1 mmol with regard to malyl units), NHS (0.2 mmol) and EDC (0.2 mmol) were mixed in 10 mL of DMSO, and stirred at RT for 2 h to form partly active carboxyl groups (NHS-PMLA-CPT). Then, mPEG_2000_-NH_2_ (0.1 mmol in 5 mL of DMSO) was added and stirred overnight at RT. The solution was dialyzed (MWCO: 8.0 kDa) against the deionized water for 24 h at 4°C and lyophilized to give P1 as khaki powder. Different grafting ratio CPT (or PEG) in P1 were synthesized by adding different proportions of CPT (or mPEG_2000_-NH_2_).

### Synthesis of FA-PEG-PMLA-CPT-I (FP1)

NHS-PMLA-CPT (1 mmol with regard to malyl units), mPEG_2000_-NH_2_ (0.098 mmol) and FA-PEG_2000_-NH_2_ (0.002 mmol) were mixed in 10 mL of DMSO and stirred overnight. The purification procedure was the same as that of P1.

### Synthesis of FITC-labeled FA-PEG-PMLA-CPT-I (FITC-labeled FP1)

NHS-PMLA-CPT (1 mmol with regard to malyl units), mPEG_2000_-NH_2_ (0.096 mmol), FA-PEG_2000_-NH_2_ (0.002 mmol) and FITC-PEG_2000_-NH_2_ (0.002 mmol) were mixed in 10 mL of DMSO and stirred overnight under a lucifugal condition. The purification procedure was the same as that of P1.

### Synthesis of PEG-PMLA-CPT-II (P2)

PMLABz (0.1 mmol), NHS (0.12 mmol) and EDC (0.15 mmol) were mixed in 10 mL of DMSO, and stirred at RT for 2 h to activate terminal carboxyl groups of PMLABz. Then, mPEG_2000_-NH_2_ (0.1 mmol in 5 mL of DMSO) was added and stirred overnight. The solution was dialyzed (MWCO: 8.0 kDa) against deionized water for 24 h at 4°C and lyophilized to obtain amphiphilic copolymers PEG-PMLABz.

Benzyl groups were taken off from PEG-PMLABz by hydrogenation to give PEG-PMLA. PEG-PMLA (1 mmol with regard to malyl units), EDC (0.5 mmol), DMAP (0.1 mmol), CPT (0.4 mmol) were mixed into 10 mL of DMSO, stirred at RT overnight. The solution was dialyzed (MWCO: 3.5 kDa) against DMSO for 24 h to remove unreacted CPT and other small molecules, then dialyzed (MWCO: 3.5 kDa) against deionized water for 24 h at 4°C and lyophilized to give P2 as khaki powder.

### Synthesis of FA-PEG-PMLA-CPT- II (FP2)

PMLABz (0.1 mmol), NHS (0.12 mmol) and EDC (0.15 mmol) were mixed into 10 mL of DMSO, and stirred at RT for 2 h to activate terminal carboxyl groups of PMLABz. Then, mPEG_2000_-NH_2_ (0.098 mmol) and FA-PEG_2000_-NH_2_ (0.002 mol) was added and stirred overnight. The hydrogenation and conjugation of CPT were the same as described above in P2 synthesis.

### Structural analysis of synthesized polymers

Polymers were dissolved in DMSO-d6 (10 mg/mL). The ^1^H-NMR spectra were obtained by Bruker Avance 500 MHz spectrometer (Germany), and the chemical shifts were expressed in ppm relative to the resonance of the internal standard, tetramethyl silane (TMS). Fourier transform infrared spectroscopy (FTIR) was also used to confirm the structure of polymers. Molecular weights and polydispersity index (PDI) of the polymers were determined by a GPC. Phosphate buffer solution (100 mM, pH 6.0) was used as a mobile phase at a flow rate of 1.0 mL/min, and a column thermostat was maintained at 25°C. Molecular weight calibration was performed with the polystyrene standards (Mp 1–30 k) from Fluka. The grafting ratio of CPT in P1 or P2 was determined by UV at 365 nm.

### Preparation of polymeric micelles

The P1 micelles were prepared by a diafiltration method. Briefly, 10 mg of P1 were dissolved in 2 mL of DMSO. Subsequently, 0.5 mL deionized water was added dropwise into the solution. The resulting solution was stirred for 0.5 h and then transferred into a pre-swollen dialysis membrane (MWCO: 3.5 kDa) and dialyzed against deionized water at 4°C. The outer phase was replaced with fresh deionized water at 1, 2, 4, 6, and 12 h. After 24 h, the dry P1 micelles were obtained by lyophillization and stored at 4°C.

The preparations of FP1 micelles, FITC-labeled P1 micelles, FITC-labeled FP1 micelles, P2 micelles, FP2 micelles, FITC-labeled P2 micelles and FITC-labeled FP2 micelles were similar to the procedures of P1 micelles.

### Dynamic light scattering (DLS)

As described in detail previously [27], the particle size and zeta potential of the self-assembled micelles were measured by a Malvern Zetasizer, MODEL NANO ZS (Malvern Instruments Limited, UK). Water dispersion (1 mL) of micelles was analyzed in a polystyrene cell at 25°C, using a He-Ne laser -wavelength of 633 nm and a detector angle of 90°. The particle size and zeta potential analysis were performed at 0.1 mg/mL in PBS (pH 7.4) at 25°C.

### Transmission electron microscope (TEM)

As described in detail previously [27], the morphology of micelles was observed by transmission electron microscopy (TEM) (JEM-2000EX, JEOL Ltd. Japan). Samples were dropped onto a copper grid coated with a carbon membrane and stained by phosphotungstic acid (2 wt% aqueous solution), then dried under vacuum before characterization.

### Critical Micellar Concentration (CMC) measurement

The critical micelle concentration (CMC) of the micelles was estimated by surface tension measurement. Briefly, a freeze-dried micelle sample was dispersed in PBS (pH 7.4) at a range of concentrations from 1×10^−4^ mg/mL to 1.0 mg/mL. Surface tension was measured at 25°C by the Wilhelmy plate method using a FACE Surface Tensiometer CBVP-A3 (Kyowa Kaimenkagaku Co. Ltd., Japan). Measurements were made 10 min after the plate was set on the liquid surface. CMC of micelles was estimated from the inflection points in the surface tension versus micelles concentration curves.

### *In vitro* drug release profile

The *in vitro* drug release profile was determined by a dynamic dialysis method. Briefly, 5 mL of CPT loaded micelles suspension (2 mg/mL) was dialyzed (MWCO: 3.5 kDa) against 100 mL PBS solution with 0.5% Tween 80 (pH 7.4 or 5.0) at 37°C. At given intervals, 200 μL of external buffer was collected and replaced by the same volume PBS solution. The concentrations of CPT released from micelles were quantified by ultraviolet spectrophotometry at 365 nm.

### Flow cytometry study

FR-positive Hela cells were maintained in a 6-well plate at 5 × 10^5^ cells/well for 24 h, and then treated with FITC-labeled micelles for 2 h at 37°C. After incubation, cells were washed with PBS three times to remove unbound micelles. The cells were then harvested by trypsinization and centrifuged at 1000 rpm for 5 min, resuspended in 500 μL PBS medium and examined by flow cytometry using a FACScan instrument (Becton Dickinson, USA).

### Confocal laser scanning microscopy observation

As described in detail previously [28], confocal fluorescent microscopy was used to confirm the uptake of micelles by FR-positive Hela cells. Similar to flow cytometry, the Hela cells were seeded into glass-bottomed dishes at a density of 2×10^5^ cells/well and incubated for 24 h at 37°C. Then, the cells were treated with various FITC-labeled micelles in fresh culture medium at 37°C for 15min, 1 or 4 h, respectively. The concentration of FITC was 1 μg/mL. Then, the cells were washed with PBS three times and fixed in precooled acetone (-20°C) for 20 min, stained with PI nuclear stain (5 μg/mL) for 5 min at 4°C in the dark. After the cells were washed with PBS solution, fluorescent images of cells were analyzed by using a FV1000 confocal microscope (Olympus, Japan).

### In vitro cytotoxicity assay

The in vitro cytotoxicities of free CPT, P1, FP1, P2 and FP2 micelles were investigated by MTT assay. FR-positive Hela cells were cultured in the RPMI-1640 medium supplemented with 10% FBS, 100 U/mL penicillin and 100 mg/mL streptomycin. The cells were seeded into 96-well plates at a density of 2×10^3^ cells/well and incubated for 24 h at 37°C 5% CO_2_ to allow cell attachment. The culture medium was then replaced by 200 μL RPMI-1640 medium containing various concentrations of the following substances: free CPT dissolved in DMSO, P1 micelles, FP1 micelles, P2 micelles or FP2 micelles. After incubating for another 48 h, the MTT solution (5 mg/mL, 20 μL) was added to each well and the cells were incubated for an additional 4 h at 37°C. Finally, the supernatant was removed and DMSO (200 μL) was added to each well to dissolve the MTT formazan crystals. The absorbance of each well was measured by the microplate reader (BIO-RAD 680, USA) at 490 nm.

### Statistical analysis

Statistical analysis was conducted by using the one-way ANOVA with Student’s *t*-test with *P*<0.05 as significant difference.

## Results and Discussion

### Preparation and characterization of polymers

**Fig 2:**
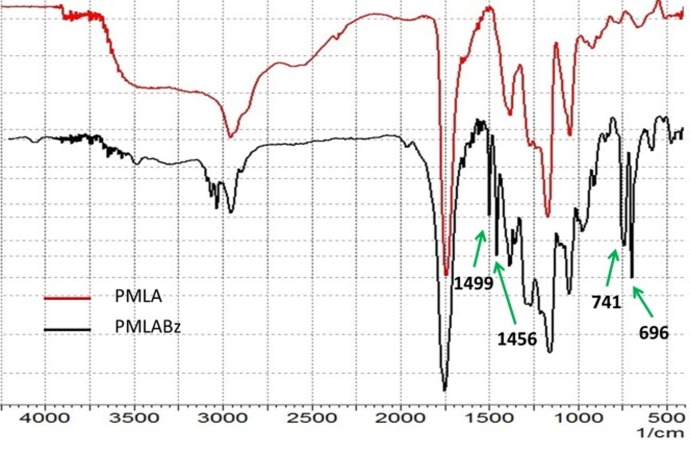
FTIR spectra of PMLA and PMLABz

**Fig 3:**
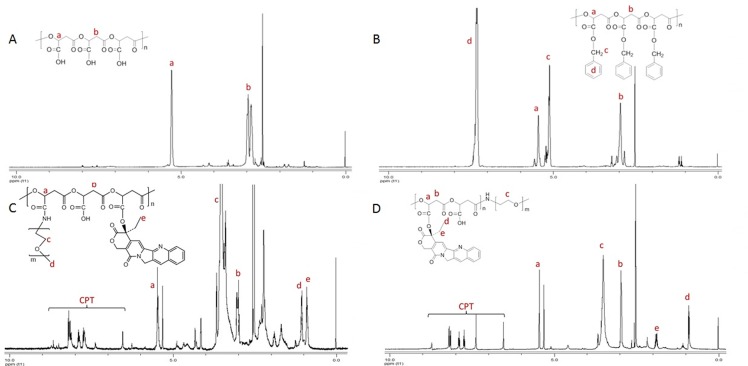
^1^HNMR spectra of PMLA(A), PMLABz(B), P1(C) and P2(D) (DMSO-d6, 500 MHz).

The PMLA was firstly synthesized by Vert M[29]. However, the applications of PMLA were restricted because of the low yield (10%-15%) of benzyl-β-malolactonate (MLABz, the key intermediate in PMLA synthesis). In our previous studies, the synthesis process of PMLA was optimized and the yield of MLABz was increased to 30%[15,28]. PMLABz was synthesized by a ring opening polymerization to control the molecular weight and chain end-groups (-COOH). Then, PMLA was obtained via the removal of benzyl groups from PMLABz by atmospheric hydrogenation reaction. The structures of the PMLABz and PMLA were characterized by FTIR (Fig 2) and ^1^HNMR (Fig 3A and 3B). As shown in Fig 2, after taking off benzyl groups, characteristic peaks of benzene ring at 741 cm^-1^, 698 cm^-1^ and 3030–3080 cm^-1^ were disappeared; meanwhile, a wide peak of hydroxy groups at 2500–3700 cm^-1^ was observed. As shown in Fig 3, the chemical shifts corresponding to both the PMLABz (2.92 –CHCH_2_CO–, 5.17 –COCH_2_C_6_H_5_–, 5.38 –OCHCH2–, 7.27 –CH_2_C_5_H_6_) and PMLA (2.92 –CHCH_2_CO–, 5.38 –OCHCH2–) were observed, which further confirmed the structure of PMLABz and PMLA. The molecular weight of PMLABz (*Mw* 15.2 kDa, Polydispersity 1.23) and PMLA (*Mw* 7.6 kDa, Polydispersity 1.16) were determined by GPC (THF, polystyrene standards, 1 mL/min), which was in accordance with the theoretical values.

**Fig 4:**
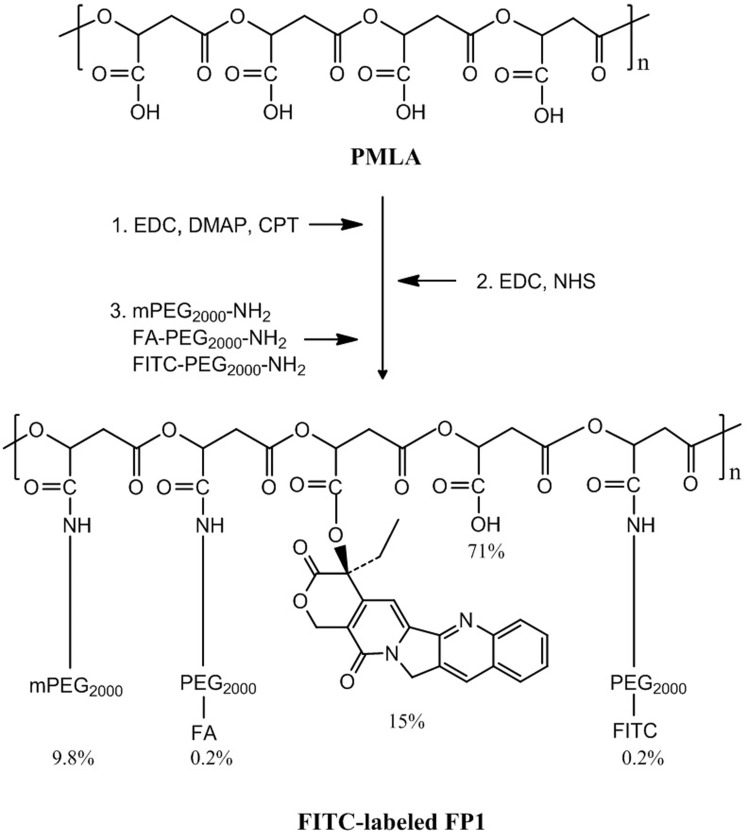
Synthetic route of FITC-labeled FP1

**Fig 5:**
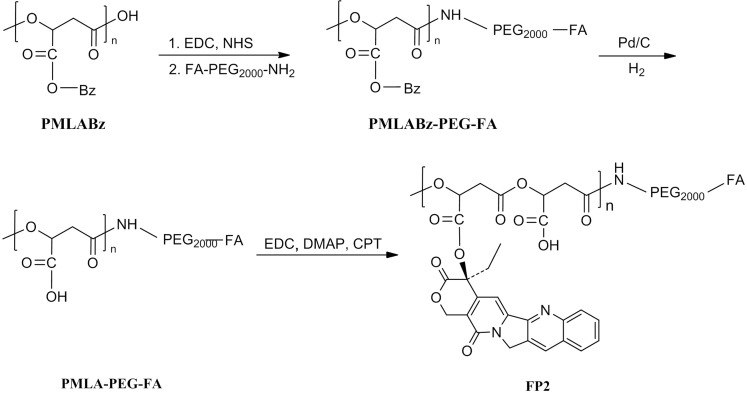
Synthetic route of FP2

P1, FP1, FITC-labeled FP1 was synthesized by controlled chemical coupling of macromolecular groups with the PMLA scaffold (Fig 4). Firstly, the scaffold carboxyl groups were partly conjugated with CPT by the ester bond. Then the polymer was reacted with N-hydroxysuccinimidyl esters to form the polymer (CPT-PMLA-NHS), followed by conjugation with mPEG_2000_-NH_2_, FA-PEG_2000_-NH_2_ and FITC-PEG_2000_-NH_2_. In the synthesis process of P2 and FP2 (Fig 5), the copolymer (FA-)PEG-PMLABz was firstly synthesized, hydrogenated off benzyl groups of PMLABz and combined CPT with the PMLA scaffold. To avoid CPT releasing during the purification step, which may happen, the copolymer was dialyzed at 4°C in deionized water. The structures of P1 and P2 were characterized by ^1^HNMR (Fig 3C and 3D).

### Characterization of different micelles

To explore the influence of grafting ratio of CPT or PEG on P1 micelles formation, various P1 micelles with different grafting ratio of CPT or PEG were prepared. Their characterization was shown in Table 1. The grafting ratio of PEG in P1 was confirmed by ^1^HNMR and the grafting ratio of CPT in P1 was determined by UV spectrophotometry at 365nm (A = 0.0655C + 0.0321, R^2^ = 0.9999, 1–10 μg/mL). Micelles were not formed in Group A because the high grafting ratio of CPT caused high hydrophobicity of the whole chain, resulting in precipitation in the dialysis. Micelles were not formed in Group D either, because the chains were free in the aqueous solution.

**Table 1:** The size and zeta potential of P1 micelles with different grafting ratio of CPT/PEG.

	PEG(5 mol%)-PMLA-CPT				PEG(10mol%)-PMLA-CPT				PEG(20 mol%)-PMLA-CPT			
	CPT	Size*	PDI	ξ	CPT	Size*	PDI	ξ	CPT	Size*	PDI	ξ
	(mol%)	(nm)		(mV)	(mol%)	(nm)		(mV)	(mol%)	(nm)		(mV)
**A**	36.31	--	--	--	35.64	--	--	--	34.74	--	--	--
**B**	21.28	96.4±6.2	0.149	-16.4	23.27	97.2±4.6	0.063	-13.2	26.91	103.2±7.1	0.106	-10.4
**C**	10.67	102.7±5.1	0.197	-22.7	15.16	114.5±5.3	0.075	-18.1	19.86	125.3±6.6	0.172	-16.2
**D**	4.95	--	--	-28.5	5.87	--		-24.8	7.39	--	--	-20.3

PDI, polydispersity index; ξ means zeta potential. Both size and zeta potential were determined in PBS (pH 7.4, 10 mM) with the concentration of 0.1 mg/mL at 25°C.

*Each value indicates the Mean ± standard deviation (SD) (nm) of three experiments.

The zeta potential of PMLA was -32 mV. The zeta potential of P1 micelles was increased with the increase of the grafting ratio of CPT or PEG, but still remains negative as showen in Table 1, which was in favor of long circulation *in vivo*.

**Fig 6:**
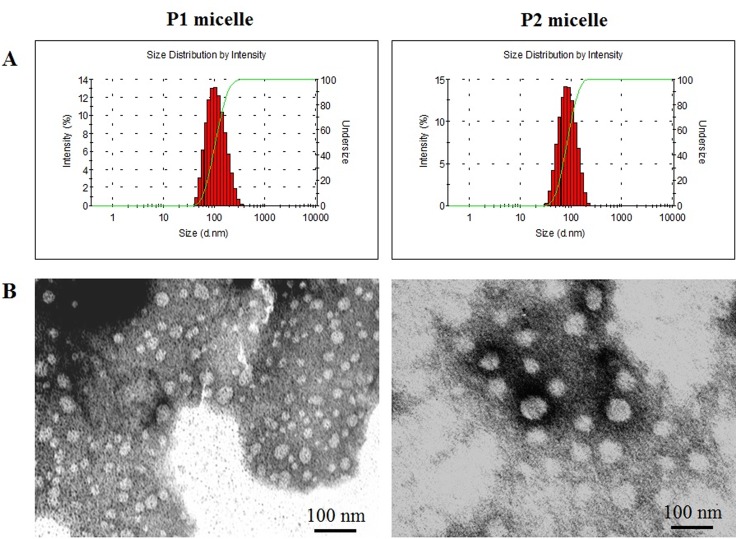
Size and morphology of P1 and P2 micelles. Size distribution of P1 and P2 micelles was determined by DLS (C = 0.1 mg/mL) (A), and morphology was observed by TEM(B).

As shown in Fig 6, for the star-like P1 micelle, the diameter was much longer in DLS analysis (97 nm) than that of observed in TEM (30–50 nm). This is probably because the data obtained in DLS analysis were hydrodynamic diameter in PBS solution and the PEG molecules were stretched out, while in TEM analysis micelles were dried and the PEG molecules were folded around the core. For the crew cut P2 micelle, the diameter from DLS analysis (76 nm) was roughly the same as that in TEM analysis (50–80 nm). This is probably because the large core was scarcely influenced by the shell. The micelles sizes at various concentrations were further measured by DLS analysis. As a result, the diameter of P1 micelles was increased dramatically from 97 nm to 800 nm when the concentration decreased 10 times, while the diameter of P2 micelles showed no significant change under the same variation. This suggested that P2 micelles have higher stability than that of P1 micelles and are more suitable for *in vivo* experiment.

The CPT loading efficiency, particle sizes at pH 7.4, zeta potential and CMC of P1, P2, FP1 and FP2 micelles were represented in Table 2. As a note, the grafting ratio of PEG in P1 and FP1 were 10 mol%. The CMC, as one of the most important physical parameters of surfactants, is the concentration in which a surfactant starts to aggregate and form micelle. The CMC of micelles were determined by surface tension measurement. The CMC of P1 and FP1 micelles were much higher than that of P2 or FP2 micelles. This further confirmed that the stability of P2 micelles is better than that of P1 micelles.

**Table 2:** The drug loading, size, zeta potential and CMC of P1, P2, FP1 and FP2 micelles.

	CPT/wt%	Size/nm*	ξ/mV	CMC/μg/mL
**P1 micelle**	11.2	97.2±4.6	-18.5	47
**P2 micelle**	20.5	76.4±3.8	-16.4	5.3
**FP1 micelle**	10.4	105.3±3.9	-20.4	56
**FP2 micelle**	19.7	82.1±2.3	-18.7	4.9

*Each value indicates the Mean±SD (nm) of three experiments obtained by DLS analysis.

### In vitro drug release studies

**Fig 7:**
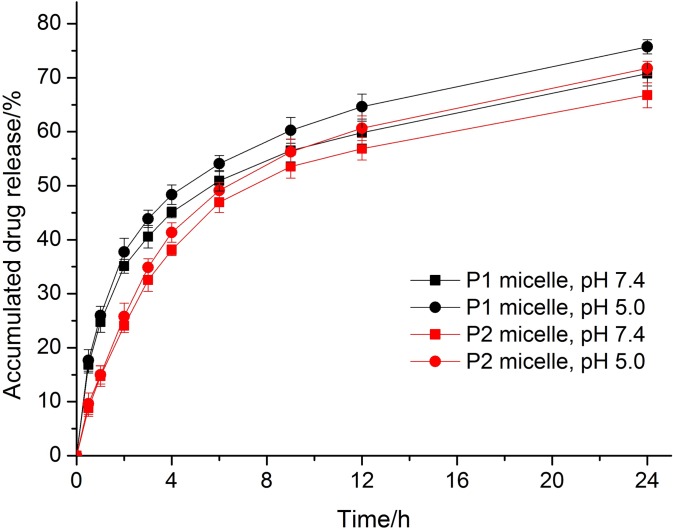
The release profile of CPT from P1 and P2 micelles *in vitro*. The release profile of CPT was measured at 37°C in PBS (pH 7.4 or 5.0, 10mM). Each value represents the Mean ± SD of three independent experiments.

Fig 7 showed the in vitro pH-dependent release behaviors of P1 and P2 micelles in two different pH buffer solutions (pH 5.0 and 7.4). As shown in Fig 7, the drug release rate in pH 7.4 was just a little slower than that in pH 5.0 for both P1 micelles and P2 micelles, which indicated that these two types of micelles do not have obvious pH-sensitivity ability due to the stability of ester bond in their structure. Moreover, the drug release profiles showed a burst-release of about 50% drugs during the first 6 h of incubation, which could be due to the reason that hydrolysis of ester bond between the CPT and PMLA scaffold was easier to be hydrolysed than phenyl ester bond (NK012 reported by Fumiaki Koizumi). Compared to P2 micelles, the P1 micelles showed a higher burst-release property, which showed up to 25% and 36% in drug release percentage in 1 h and 2 h respectively, while P2 micelles showed only 15% and 24%.

### Cellular uptake and flow cytometry study

**Fig 8:**
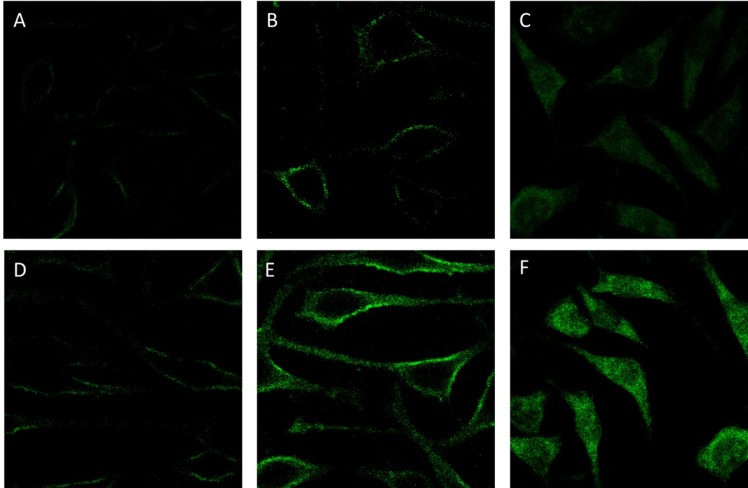
Cellular uptake of FITC-labeled P1 micelles and FP1 micelles in FR-positive Hela. Cells were incubated with FITC-labeled P1 micelles for 15 min (A), 1 h (B) or 4 h (C) at 37°C. Cells were incubated with FITC-labeled FP1 micelles for 15 min (D), 1 h (E) or 4 h(F) at 37°C. Then the Cellular uptake was observed by confocal laser scanning fluorescence microscopy.

**Fig 9:**
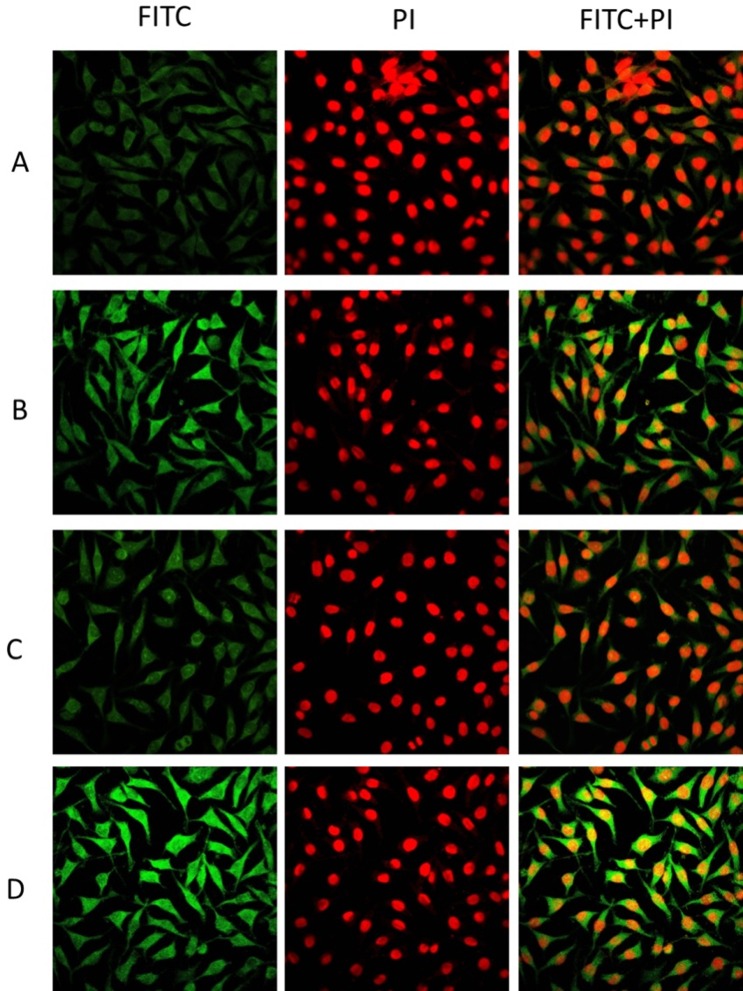
Cellular uptake of micelles in FR-positive Hela cells. Cells were incubated with (A) FITC-labeled P1 micelles, (B) FITC-labeled FP1 micelles, (C) FITC-labeled P2 micelles, (D) FITC-labeled FP2 micelles for 4 h at 37°C. The Cellular uptake was observed by confocal laser scanning fluorescence microscopy. The green fluorescence and red fluorescence represent the localization of FITC and PI, respectively.

The cellular uptake and intracellular transport of P1, P2, FP1 and FP2 micelles were observed with CLSM (Figs 8 and 9). P1 micelles and P2 micelles were negatively charged, which was unfavorable for endocytosis. In order to enhance endocytosis, FA was introduced as a ligand. As shown in Fig 8, FR-positive Hela cells were incubated with FITC-labeled P1 micelles or FITC-labeled FP1 micelles for 15 min, 1 h and 4 h, respectively. FP1 micelles showed much higher cellular accumulations than that of P1 micelles, which might be due to the FA-mediated enhanced cellular uptake. But no significant difference was observed between P1 micelles and P2 micelles in cellular uptake (Fig 9), similar result was obtained between FP1 and FP2 micelles. Here, a red nuclear dye was chosen to visualize cell nuclei.

**Fig 10:**
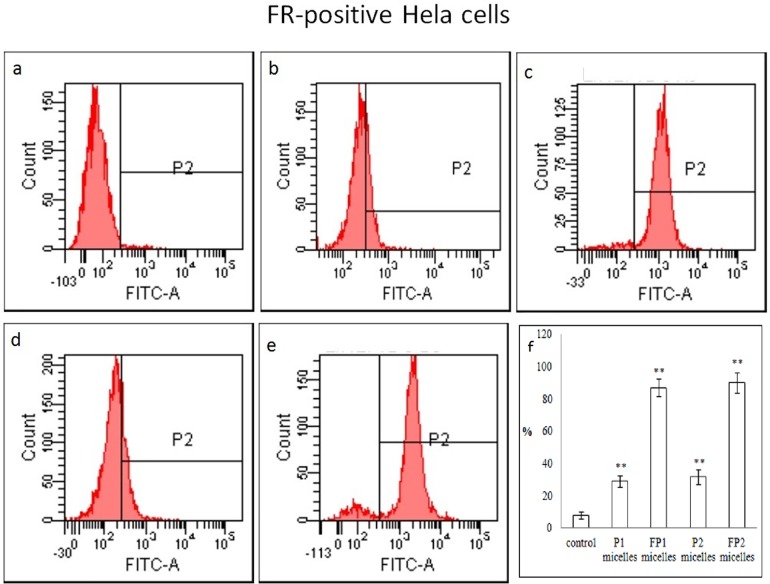
Flow cytometry analysis of cellular uptake in FR-positive Hela cells. Cells were incubated with micelles for 2 h at 37°C before flow cytometry analysis. (a) control, (b) FITC-labeled P1 micelles, (c) FITC-labeled FP1 micelles, (d) FITC-labeled P2 micelles, (e) FITC-labeled FP2 micelles. (f) Data were presented as Mean ± SD (n = 3). *P < 0.01 vs control. Student’s *t*-test.

The flow cytometry profiles of FR-positive Hela cells incubated for 2 h with FITC-labeled P1 micelles, FITC-labeled FP1 micelles, FITC-labeled P2 micelles or FITC-labeled FP2 micelles were shown in Fig 10. The amount of samples internalized by the cells was proportional to the fluorescence intensity of FITC. The cellular uptake was much higher in FA-modified micelles than that in non-FA-modified micelles. This suggested that FA-targeted micelles could enhance the cellular uptake by a folate receptor-mediated endocytosis process, which is in agreement with the results of CLSM. In addition, there was no significant difference between P1 micelles and P2 micelles in cellular uptake.

### In vitro cytotoxicity

**Fig 11:**
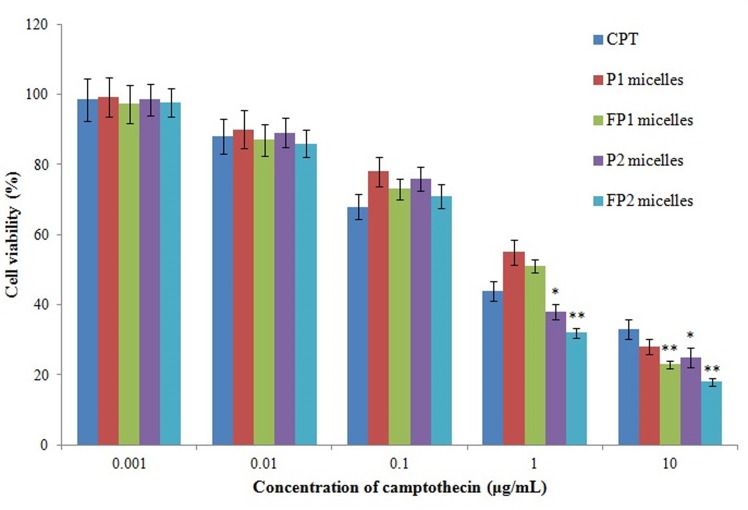
Cell cytotoxicity of micelles and free CPT in FR-positive Hela cells. Free CPT, P1 and FP1 micelles, P2 and FP2 micelles were incubated with cells for 48 hours. The cytotoxicity was determined by MTT assay. Each value represents the Mean ± SD of three independent experiments.

In vitro antitumor activities of free CPT and P1, P2, FP1, FP2 micelles against FR-positive Hela cells were studied by MTT assay (Fig 11), and median inhibitory concentration (IC_50_) was determined (Table 3). As shown in Fig 11, all micelles showed concentration-dependent toxicity. At 10 μg/mL of CPT, all 4 kinds of micelles showed higher cytotoxicity than that of free CPT (*P*<0.05 or *P*<0.01). This result suggested that micelles could increase the cellular uptake, especially the FA-modified micelles. In addition, P2 and FP2 micelles still showed higher cytotoxicity than that of free CPT at 1 μg/mL of CPT (*P*<0.05 or *P*<0.01), while P1 and FP1 micelles showed lower cytotoxicity than that of free CPT. This is probably because the particle size of P1 and FP1 micelles increased dramatically when the concentration decreased from 10 μg/mL to 1μg/mL, while the particle size of P2 and FP2 micelles could keep constant under the same variation. These results suggested that P2 and FP2 have better stability than that of P1 and FP1. The IC_50_ data also confirmed theses results (Table 3).

**Table 3:** IC_50_ values for CPT and micelles in FR-positive Hela cells. (Mean ± SD, n = 3).

	IC_50_ (μg/mL)*
CPT	0.822±0.032
P1 micelles	1.268±0.056
FP1 micelles	0.703±0.013
P2 micelles	0.538±0.028
FP2 micelles	0.310±0.011

* IC_50_ values were represented as concentration of CPT.

## Conclusion

PMLA is a novel drug delivery carrier with many promising advantages. However, the high water-solubility of PMLA holds up its applications to some extent. In this paper, a novel strategy was developed to reduce the water-solubility of PMLA by introducing CPT to PLMA scaffold. At the same time, the water-solubility of poorly soluble CPT could be tremendously improved. To find a good connection way of CPT to PLMA, two types of biodegradable CPT-conjugated polymeric micelles were prepared based on PMLA derivatives. The core of P1 and P2 micelles loaded abundant CPT by covalent bonds. Meanwhile, the hydrophilic/hydrophobic property of PMLA was effectively adjusted by changing the grafting ratio of CPT as a hydrophobic segment, and finally formed stable micelles after optimization. Though P1 and P2 micelles were both nano-sized and could improve the cellular uptake of CPT, P2 micelles (formed by amphiphilic block copolymer) showed higher stability, higher drug loading efficiency, smaller size, and slower drug release rate than that of P1 micelles (formed by grafted copolymer). These observations offer the potential use of P2 micelles for cancer therapy.
